# Nasal lavage cytology and mucosal histopathological alterations in patients with rhinitis^☆^

**DOI:** 10.1016/j.bjorl.2019.01.005

**Published:** 2019-02-22

**Authors:** Loreni C.S. Kovalhuk, Ederaldo Queiroz Telles, Monica Nunes Lima, Nelson A. Rosario Filho

**Affiliations:** a Universidade Federal do Paraná (UFPR), Programa de Pós-Graduação em Saúde da Criança e do Adolescente, Curitiba, PR, Brazil; b Universidade Federal do Paraná (UFPR), Curitiba, PR, Brazil

**Keywords:** Allergic rhinitis, Eosinophil, Basement membrane, Airway remodeling, Rinite alérgica, Eosinófilo, Membrana basal, Remodelamento da via aérea

## Abstract

**Introduction:**

The extent of epithelial lesion in allergic and non-allergic rhinitis and its association with inflammatory changes in nasal lavage has not been clarified.

**Objective:**

To verify the association between the inflammatory cells in the nasal lavage, epithelial lesion extent and basement membrane thickness, in the nasal mucosa of patients with rhinitis; to determine the cutoff point of the percentage of eosinophils in the nasal lavage associated with the atopic patients.

**Methods:**

Patients with rhinitis and indication for septoplasty and (or) turbinectomy for turbinate hypertrophy were selected, and were submitted to allergy skin tests, nasal lavage with measurement of albumin and interleukin-8 levels, total and differential counting of cells, and mucosal histopathological analysis to determine the extent of epithelial lesion, and degree of basement membrane thickening.

**Results:**

Fifty-six patients with a median age of 24.5 years and a diagnosis of allergic rhinitis (*n* = 36) and non-allergic rhinitis (*n* = 20) were studied. In atopic subjects, allergy skin tests were positive for *Dermatophagoides pteronyssinus* in 35 (97.0%) and *Lolium perenne* in 18 (50.0%). Atopic subjects showed a higher clinical score index of rhinitis compared to non-atopic ones. The total count of cells, neutrophils, and levels of albumin and IL-8 were not different in the nasal lavage of atopic and non-atopic subjects. The cutoff point for eosinophil count in nasal fluid for the distinction between allergic rhinitis and non-allergic rhinitis was 4%. Some degree of epithelial lesion was more frequent in allergic rhinitis (94%) than in non-allergic rhinitis (65%) patients. In the presence of basement membrane thickness, as a marker of remodeling, there was no difference in the nasal lavage of patients with allergic rhinitis and non-allergic rhinitis.

**Conclusion:**

In this series, 4% was the cutoff point for the number of eosinophils in the nasal lavage, for atopy differentiation. Upper airway remodeling accessed by basement membrane thickness showed similar inflammatory cell infiltrate in the nasal lavage, regardless of the presence of atopy.

## Resumo

**Introdução:**

A extensão da lesão epitelial na rinite alérgica e não alérgica e sua associação com alterações inflamatórias no lavado nasal ainda não estão esclarecidas.

**Objetivo:**

Verificar a relação entre o processo inflamatório no lavado nasal, extensão da lesão epitelial e espessamento da membrana basal na mucosa nasal de pacientes com rinite; determinar o ponto de corte do percentual de eosinófilos no lavado nasal associado à presença de atopia.

**Método:**

Foram selecionados pacientes com rinite e indicação de septoplastia e (ou) turbinectomia por hipertrofia de conchas nasais, os quais foram submetidos aos testes cutâneos alérgicos, lavado nasal com determinação das concentrações de albumina, interleucina-8 (IL-8), contagem total e diferencial de células, análise da extensão da lesão epitelial, e grau de espessamento da membrana basal por meio de histopatologia da mucosa.

**Resultado:**

Foram estudados 56 pacientes, mediana de 24,5 anos com diagnóstico de rinite alérgica (n = 36) e rinite não alérgica (n = 20). Nos atópicos os testes cutâneos alérgicos foram positivos para *D. pteronyssinus* em 35 (97,0%) e *L. perenne* em 18 (50,0%). Atópicos apresentaram maior índice de escore clínico para rinite em comparação a não atópicos. A contagem total de células, neutrófilos e níveis de albumina e IL-8 não foi diferente entre o lavado nasal de atópicos e não atópicos. O ponto de corte da contagem de eosinófilos no fluido nasal para a distinção de rinite alérgica e rinite não alérgica foi de 4%. Algum grau de lesão epitelial foi mais frequente em pacientes com rinite alérgica (94%) do que em pacientes com rinite não alérgica (65%). Na presença de espessamento da membrana basal, como marcador de remodelamento, não houve diferença no lavado nasal entre pacientes com rinite alérgica e não alérgica.

**Conclusão:**

Nesta casuística, 4% foi o ponto de corte do número de eosinófilos no lavado nasal, para diferenciação de atopia. Remodelamento da via aérea superior pelo espessamento da membrana basal revelou infiltrado semelhante de células inflamatórias no lavado nasal, independentemente da presença de atopia.

## Introduction

Rhinitis is a chronic, prevalent disease with a complex integration between multiple genetic and environmental factors, interconnected by mechanisms associated or not with IgE. The association with other allergic diseases and phenotypes related to multiple allergen sensitization influences the intensity, frequency and persistence of symptoms.^1^ The chronic inflammatory process in the respiratory mucosa can lead to structural alterations with airway remodeling, well characterized in asthmatic patients,^2^, ^3^, ^4^ but to a lesser extent in those with rhinitis.^5^, ^6^, ^7^, ^8^, ^9^, ^10^, ^11^All inflammatory diseases result in remodeling, which can progress to a normal or pathological reconstruction process.^4^ In rhinitis, it is characterized by increased thickness and epithelial detachment and pseudofibrosis of the basement membrane.^10^

The inflammatory reaction and the remodeling of the nasal turbinate mucosa results in turbinate edema and (or) hypertrophy, of which clinical consequence is nasal obstruction.^9^, ^12^ Additionally, the variations in the engorgement of the complex arterial vasculature and of cavernous venous sinusoids also contribute to severe nasal obstruction.^3^, ^13^ An increase in the number of eosinophils in the nasal mucosa is the parameter that shows the best correlation with the nasal obstruction symptom.^12^

The extent of the epithelial lesion in the different types of rhinitis and the correlation with inflammatory cells and mediators is yet to be clarified. The presence of eosinophils is associated with loss of epithelial integrity in patients with allergic or non-allergic rhinitis.^14^ However, there are disagreements regarding the cutoff point for the number of eosinophils considered high in nasal secretion samples, since the nasal secretion collection procedure can interfere with the recovered cellularity.^15^, ^16^, ^17^ The nasal mucosa, due to the easily obtained samples, allows the study of cell alterations during the allergic reaction.^15^ Nasal lavage is a relatively noninvasive and easy to perform technique for the quantitative measurement of cell distribution and inflammatory mediators.^3^, ^18^, ^19^, ^20^ The simultaneous analysis of the cell infiltrate and the degree of cell activation of nasal secretion and nasal mucosa biopsy samples shows differences between the two compartments.^21^ Thus, the study of the inflammatory process in samples simultaneously obtained by nasal lavage (NL) and from the nasal mucosa may help to understand the mechanisms involved in allergic or non-allergic nasal inflammatory reaction.

The aim of this study was to evaluate the association between the inflammatory process in the nasal cavity lumen and the extent of the nasal mucosa lesion in atopic and nonatopic patients with rhinitis.

## Methods

Patients with allergic or nonallergic obstructive rhinitis, with surgical indication for septal deviation correction and (or) turbinectomy to alleviate nasal obstruction, were selected at the Otorhinolaryngology Service of Hospital de Clínicas of Universidade Federal do Paraná. Patients with a history of upper and lower airway infection, respectively, at 2 and 4 weeks prior to clinical evaluation were excluded; in addition to patients with a history of recent use of medications such as systemic and/or nasal topical corticosteroids, disodium cromoglycate and oral and/or nasal topical antihistamines.

The clinical diagnosis and rhinitis severity classification were attained using a symptom-adjusted (pruritus sneezing, nasal obstruction, nasal secretion/sniffing, post-nasal secretion/snorting) and sign-adjusted score (mucosa color and turbinate volume increase, aspect and volume of nasal secretion, presence of alterations at the oroscopy), graded on a scale going from 0 to 3 (absent, mild, moderate and severe), with a maximum score of 24.^3^, ^22^, ^23^

The patients underwent skin prick tests,^3^, ^24^ using the extracts of the most relevant allergens in the city of Curitiba, state of Parana, Brazil,^25^, ^26^, ^27^ such as *Dermatophagoides pteronyssinus* 5.000 BAU (bioequivalent allergen unit)/mL and *Lolium perenne* (10.000 BAU/mL), obtained from Hollister-Stier®, Spokane, USA.

The test was considered positive if the mean diameter of the papule was ≥3 mm with an erythema halo, in relation to the negative control.^3^, ^24^

The patients were submitted to nasal instillation of 5 mL of isotonic saline solution (0.9%) in both nasal cavities to obtain NL fluid,^19^, ^20^, ^21^, ^28^ which was homogenized by shaking and centrifuged to obtain the cell pellet (1000 rpm/5 min) in a refrigerated centrifuge, so that only 0.01% of the cells remain in the supernatant.^18^

The supernatant was stored at −80 °C for the measurement of mediators. The albumin concentration in the nasal lavage sample was determined by a turbidimetric immunoassay (Microbumin MULTIGENT – Abbott Laboratories of Brazil Ltda®, detection limits of 1–500 μg/mL); the level of interleukin-8 (IL-8) was measured by quantitative enzyme-linked immunosorbent assay (ELISA MAX™ Set Deluxe – Biolegend®, San Diego, California, detection limits of 31.2–2000 pg/mL).

The nasal cytology analysis of the initially obtained pellet (cell pellet) allowed the determination of the total number of cells/mL, inflammatory and epithelial cell counts; an aliquot was cytocentrifuged (LABHO® CT-12 Cytocentrifuge) for the preparation of slides that were stained by the May–Grünwald–Giemsa method for differential counting of eosinophils, neutrophils and epithelial cells.^18^, ^19^, ^20^, ^29^

The histopathological analysis of mucosa samples from the inferior nasal turbinates, obtained by partial turbinectomy procedure of the inferior nasal turbinate^30^ or by mucosal biopsy of the anteroinferior tip of the inferior turbinates, was evaluated by light microscopy.^21^

The anterior portion of the inferior turbinate, more exposed to airflow and to the greater impact of aeroallergens and irritants, is more representative for the evaluation of the nasal mucosa inflammatory process^31^ if the largest amount of tissue and mucus is collected to minimize damage to the sample.^30^, ^31^ The sample was fixed in formaldehyde and the tissue block was paraffin-embedded for later cutting and slide preparation.^8^, ^32^ The stains used were: Hematoxylin-Eosin (HE) to identify leukocytes, Periodic Acid-Schiff (PAS) to help identify goblet cells and trichrome of Gomori to help identify and measure the subepithelial collagen thickness, according to the routine of the Anatomopathological Service of Hospital de Clínicas-UFPR.

The areas of epithelium preferentially covered with mucus were evaluated, excluding fields with evidence of iatrogenic detachment of the epithelium and presence of extravasated red blood cells. Aiming to avoid false results caused by the cut tangency, the image and position of the cell nuclei were evaluated.^33^

The staging of the Epithelial Lesion (EL) and the degree of basement membrane thickening were based on the score proposed by Ponikau et al.^32^ The epithelial lesion degree staging ranged from 0 to 3 (0: intact epithelium, 1: absence of ciliated cells, 2: erosion of the upper layer of cells with intact basement cell layer, 3: complete epithelium erosion), whereas the staging of the basement membrane thickening ranged from 0 to 2 (0: basement membrane not visualized; 1: visible basement membrane with thickness ≤ 20 μm, 2: visible basement membrane with thickness > 20 μm).^32^

At the statistical analysis, the estimate of the difference of asymmetric distribution variables was performed by a non-parametric test (Mann–Whitney), whereas categorical variables were compared by Fisher's exact test and Pearson's Chi-square test. A minimum significance level of 5% and a minimum test power of 90% were considered for all tests. A receiver operating curve (ROC) was constructed to estimate the cutoff point with greater sensitivity and specificity. Univariate logistic regression was performed to estimate the probability of atopy according to the eosinophil count.

This study was submitted to and approved by the Human Research Ethics Committee of Hospital de Clínicas of Universidade Federal do Paraná (UFPR), registry number 755.174/2003-11.

## Results

A total of 56 patient samples were analyzed, grouped according to the presence of atopy. Allergy skin tests were negative in 20/56 (36.0%) and positive in 36/56 (64.0%). Among the atopic subjects, the skin prick test was positive for *D. pteronyssinus* in 35/36 (97.0%) and/or for *L. perenne* in 18/36 (50.0%). The test was exclusively positive for *D. pteronyssinus* in 18/36 (50%) and for *L. perenne* in only 1/36 (3%).

The median age was 24.5 years (14–58 years), with equal distribution by gender. The total symptom score was higher in atopic subjects (9 [1–18]) than in nonatopic ones (6.5 [0–12]) (*p* = 0.01).

Atopic subjects tended to show a higher frequency of pruritus and sneezing of moderate to severe intensity (47.2%), although at the limit of significance (*p* = 0.05). The frequency of the moderate to severe nasal obstruction symptom, present in 25/36 (69.0%) of atopic subjects, was comparable to nonatopic ones (*p* = 0.16).

Total and differential cell counts, as well as albumin and IL-8 levels, are shown in Table 1. The total number of NL cells and the differential count of epithelial, neutrophil and mononuclear cells were equally distributed between atopic and nonatopic subjects. Only the percentage of eosinophils was higher among atopic ones (*p* < 0.01).

**Table 1:** Total and differential cell count, albumin and IL-8 levels in nasal lavage of atopic and non-atopic subjects.

Nasal lavage	Atopic (*n* = 36)	Non-atopic (*n* = 20)	*p*
Total cellularity	127,000 (10 × 10^3^–6.134 × 10^3^)	128,000 (24 × 10^3^–682 × 10^3^)	0.90
Epithelial cells (%)	48 (8–98)	76 (10–100)	0.07
Eosinophils (%)	3 (0–66)	1 (0–21)	<0.01
Neutrophils (%)	41.5 (0–87)	17.5 (0–83)	0.24
Mononuclear cells (%)	1 (0–12)	1 (0–7)	0.96
Albumin (μg/mL)	16 (5–338)	16.5 (5–105)	0.67
IL-8 (pg/mL)	80 (30–1300)	81.5 (30–604)	0.45

Median (limits); Mann–Whitney test.

**Figure 1:**
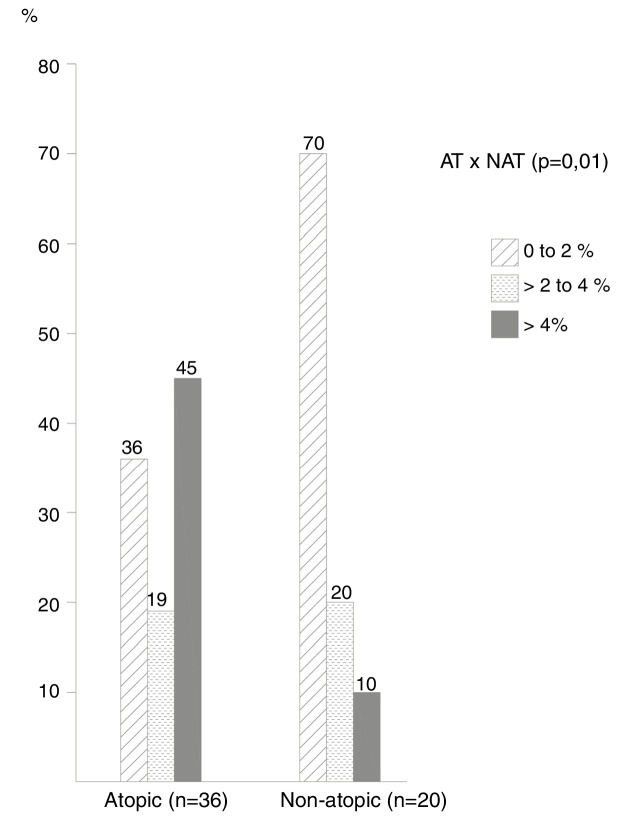
Frequency of cases distributed by percentage ranges of eosinophil numbers in nasal lavage. Chi-square test with linear trends (*p* = 0.01).

The frequency of cases by ranges of percentage of eosinophils in the NL (Fig. 1) showed that most non-atopic subjects were concentrated within the range of 0%–2%, 14/20 (70%) of the cases in relation to 13/36 (36%) of the atopic ones (*p* = 0.01). On the other hand, the frequency of cases with eosinophil counts > 4% among atopic subjects was higher, observed in 16/36 (45.0%) of these patients, compared to only 2 (10%) of non-atopic ones.

The cutoff point with the highest sensitivity and specificity indexes was 4% of eosinophils in the NL, which differentiated atopic from non-atopic subjects; with this criterion the sensitivity was 44% and the specificity was 90%. Also with a 4% cutoff, the probability of atopy was 80% (*p* < 0.001), increasing to 100% with a 10% eosinophil count.

**Figure 2:**
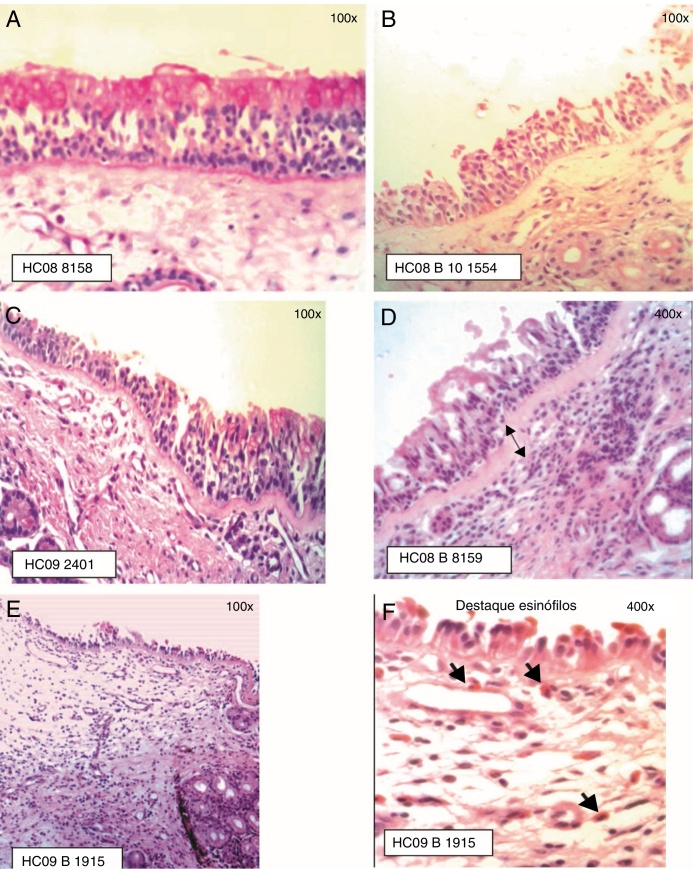
Staging of Epithelial Lesion (EL) and BM thickening: (A) EL grade 0 (intact epithelium) and BM 0 (not visualized), PAS staining; (B) EL grade 1 (absence of ciliated cells) and BM 0 (not visualized), HE staining; (C) EL grade 1 (absence of ciliated cells) and BM 1 (visible BM, thickness ≤ 20 μm), PAS staining; (D) EL grade 1 (absence of ciliated cells) and BM 2 (visible BM, thickness > 20 μm), HE staining; (E) EL grade 2 (epithelial erosion, intact basement cell layer) and BM 1 (BM ≤ 20 μm); (F) Eosinophil infiltration is highlighted, HE staining.

The ciliated columnar pseudostratified epithelium was observed in most samples (Fig. 2). In some samples, the presence in some areas of dysplastic epithelium (*n* = 1), transitional to non-keratinized squamous type (*n* = 7) was observed. The infiltrate in the chorion/lamina propria was predominantly of the mononuclear, lymphoplasmacytic type.

The presence of epithelial lesion and basement membrane thickening are shown in Fig. 2.

Among the atopic subjects, the prevalence of epithelial lesion was 94.4% and the epithelial lesion prevalence rate was 1.4 times higher (*p* < 0.01). The prevalence of basement membrane lesion was 67.0%, indicating, in the atopic subjects, the non-significant chance of having some degree of basement membrane thickening 1.2-fold higher in relation to non-atopic ones (*p* = 0.40).

**Figure 3:**
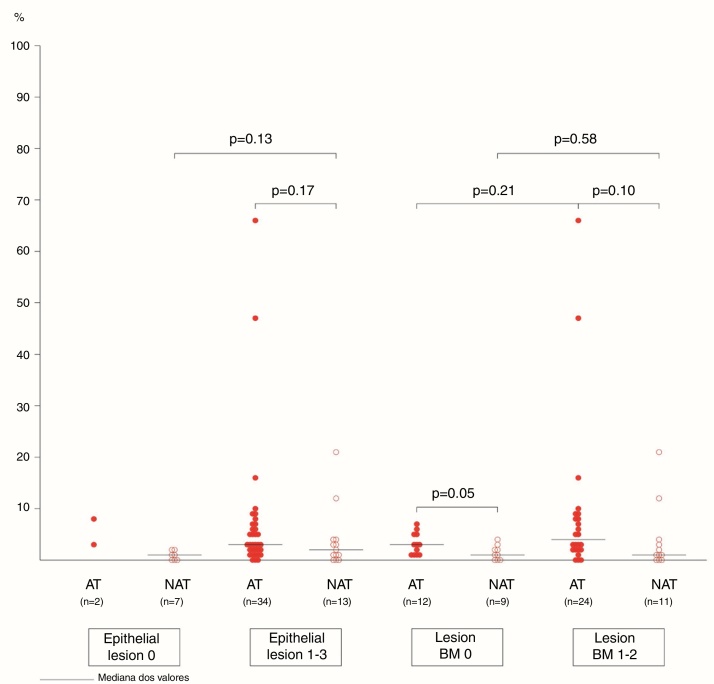
Eosinophils (%) in nasal lavage of atopic and non-atopic subjects according to epithelial lesions and basement membrane thickening. –, median values (limits); Mann–Whitney test.

**Figure 4:**
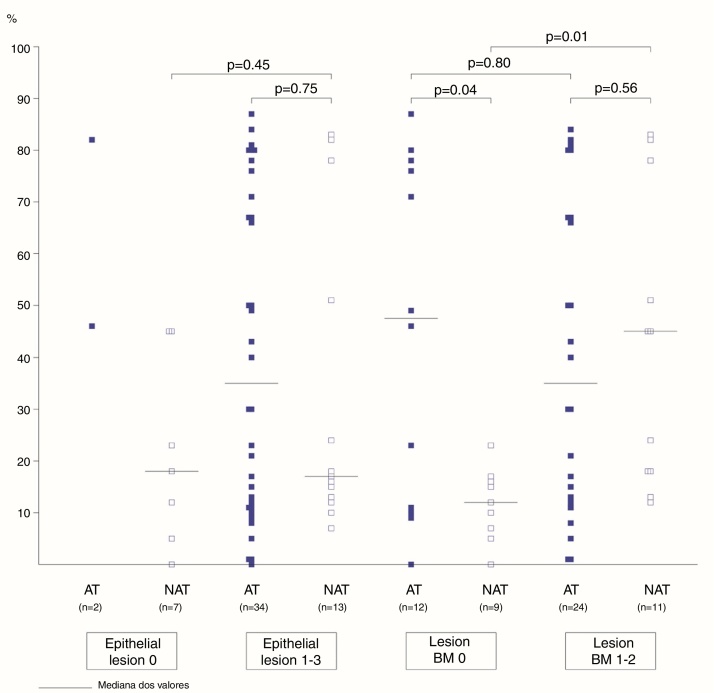
Neutrophils (%) in the nasal lavage of atopic and non-atopic subjects according to epithelial lesions and basement membrane thickening. –, median values (limits); Mann–Whitney test.

The association between the inflammatory process of the nasal cavity lumen (nasal lavage) and the nasal mucosa (histopathological analysis) showed that regardless of the atopy, in the presence of some degree of epithelial lesion, the total cell count of NL (*p* = 0.18); of eosinophils (*p* = 0.17) and neutrophils (*p* = 0.75); levels of albumin (*p* = 0.50) and IL-8 was similar (*p* = 0.09) (Figure 3, Figure 4). However, only two atopic subjects did not have histopathological epithelial lesion. On the other hand, there was no difference in cellularity and mediators in non-atopic individuals with or without epithelial lesion. Therefore, in the study sample, the presence of epithelial lesion was not associated with changes in the NL.

The difference in eosinophil count in the NL of atopic and non-atopic subjects, without BMT, was borderline (*p* = 0.05). In the presence of BMT, there were no differences in the subgroups of patients regarding the number of eosinophils in the NL. On the other hand, the BMT was associated with an increase of neutrophils in the nonatopic (NAT) subjects’ NL (*p* < 0.01). The number of neutrophils in the NL of atopic (AT) subjects without BMT was higher than in the NAT ones (*p* = 0.04). Analysis of the other NL parameters did not show significant differences in the presence of BMT (Figure 3, Figure 4).

## Discussion

Rhinitis is the consequence of an allergic and non-allergic nasal mucosa inflammatory process, associated with an accumulation of inflammatory cells in the nasal cavity lumen and structural changes in the nasal mucosa. Quantifying the extent and degree of epithelial lesion, as well as the association with different cell types in the nasal mucosa in the different types of rhinitis may help in the assessment of nasal mucosa remodeling.

Studies on the occurrence and extent of remodeling in rhinitis are conflicting, because the nasal mucosa is more exposed to external stimuli, resulting in some degree of adaptive inflammatory process or due to the different remodeling criteria used in the studies.^3^, ^5^, ^9^, ^10^, ^14^, ^15^, ^31^, ^32^, ^33^ Nasal obstruction is one of the most common and uncomfortable symptoms of rhinitis,^34^ and it is an important complaint in this group of patients, whether allergic or non-allergic ones. However, cases were selected at the Otorhinolaryngology outpatient clinic due to the surgical indication of septal deviation correction and/or turbinectomy due to turbinate hypertrophy, which are anatomical changes that also contribute to nasal obstruction.

The total score of the rhinitis score was higher in the atopic patients and, for atopy investigation, the extracts used in the skin prick test were selected according to the most relevant regional aeroallergens,^25^, ^26^, ^27^ with a sensitization frequency of 97% for *D. pteronyssinus* and 50% for *L. perenne*. Nasal provocation tests with allergens could circumvent the bias of including patients with local allergic rhinitis or sensitized to other allergens in addition to house dust mites and grass pollen in the non-atopic group.^35^

In this study, the six patients sensitized to pollen were also sensitized to *D. pteronyssinus* and their rhinitis score was not different in relation to the other atopic subjects.

The inflammatory process evaluation of the nasal mucosa, through nasal lavage and analysis by quantitative cytology of the nasal lavage, allows the assessment of the cell infiltrate and of mediators,^3^, ^18^, ^19^, ^20^ with the collection being more representative when carried out in both nostrils,^15^ which is why samples were collected from both nostrils in the present study.

The cells recovered by the nasal lavage are derived from epithelial desquamation and increased vascular permeability of the epithelium, which allows the passage of plasma proteins and inflammatory cells into the nasal cavity lumen.^15^

In this series, IL-4, IL-5 and IFN-γ levels were undetectable, possibly because the amounts of these mediators in samples collected without the allergenic stimulus of the nasal provocation test were labile and minimal. However, IL-4, IL-5 and IL-13 levels may be elevated in the nasal secretion of patients with persistent severe allergic rhinitis.^36^

The wide distribution limit of IL-8 in atopic and non-atopic NL samples may have masked any difference between the groups. This study showed no correlation between albumin levels and percentage of nasal lavage eosinophils in both groups of patients with rhinitis. This association is described in nasal provocation tests with allergens, where the increase of vascular permeability and the inflammatory cell influx is significantly higher,^37^, ^38^ as well as in patients with allergic rhinitis and asthma, who also show extensive involvement by computed tomography assessment of the paranasal sinuses.^20^

Cellular infiltration in the nasal cavity lumen, reflecting epithelial desquamation and increased mucosal permeability, was similar in this group of patients with allergic or non-allergic rhinitis; only the number of eosinophils in the NL differentiated the group of atopic subjects.

The increase in the number of nasal eosinophils show a better correlation with the nasal obstruction symptom in patients with persistent allergic rhinitis.^12^ In the study cases with moderate to severe nasal obstruction, the proportion of eosinophils in the nasal lavage was higher in the atopic group. Eosinophil infiltration is more evident after nasal provocation tests with allergens,^18^, ^38^ but there is a disagreement regarding the cutoff point of the number of eosinophils that is considered high in nasal secretion samples.

The difficulty in comparing study results is due to the different material collection methods, either by scraping, blowed secretion and different techniques of nasal lavage, as well as several methods of staining and criteria for nasal eosinophil quantification.^15^, ^16^, ^17^ In samples from the quantitative nasal lavage cytology, the 5% eosinophil cutoff resulted in an accuracy of 82% (sensitivity 80% and specificity 83%) in the diagnosis of perennial allergic rhinitis.^19^

In adults, nasal fluid eosinophil count was 6% in patients with allergic rhinitis and 2% in those with non-allergic rhinitis, and the best cutoff point was established as being 4%.^39^

In the present study, considering the 4% eosinophil cutoff point, the probability of atopy was 80% and increased to 100% with a 10% eosinophil count. The 4% eosinophil cutoff point in the nasal lavage has better sensitivity and specificity indexes when differentiating allergic from non-allergic rhinitis.

The disadvantage of the nasal lavage technique is the fact that the recovered cells originate only from the nasal cavity lumen and do not necessarily reflect the epithelial tissue inflammatory process,^40^ in which the reticular basement membrane thickening is used as a parameter for airway remodeling.^9^

Atopic subjects more frequently had some degree of epithelial lesion, but this finding was not associated with differences in the inflammatory process in the nasal cavity lumen when compared to non-atopic ones. Amin et al. reported loss of epithelial integrity associated with increased number of eosinophils, but not of neutrophils, in the nasal mucosa of patients with persistent allergic and non-allergic rhinitis.^14^

According to the findings by Lim et al., nasal secretion and nasal mucosa represent distinct compartments with different populations of leukocytes. After provocation tests with allergens, there was no epithelial destruction, basement membrane thickening or subepithelial collagen deposition, as described in bronchial asthma.^21^

Few studies have investigated the structural remodeling in the nasal mucosa of patients with allergic rhinitis and the data are conflicting.^36^ The presence and degree of basement membrane thickening may vary according to the assessed region. In the anterior region of the inferior turbinate, where the respiratory epithelium predominates, the proportion of basement membrane thickening is higher.^31^ Because it is more exposed to airflow, where there is greater impact of aeroallergens and irritants, some aspects of the inflammatory process and remodeling findings may represent an adaptive response of the nasal mucosa, making it difficult to compare patients without and with rhinitis.^14^, ^31^, ^33^

Epithelial lesion, characterized by the presence of intercellular edema, epithelial desquamation and eosinophilic clusters, was demonstrated in the anterior nasal mucosa of patients allergic to dust mites.^6^, ^14^, ^31^ In eight of the samples from the present study, areas of dysplastic epithelium and transitional to the non-keratinized squamous type were observed, also described by other authors.^41^, ^42^

An increased number of eosinophils in the submucosa of patients with persistent severe allergic rhinitis is not always accompanied by changes in epithelial integrity and/or basement membrane thickening,^36^ which may be associated with protective mechanisms that minimize remodeling and potentiate epithelial regeneration.^41^

However, when there is severe nasal obstruction due to persistent turbinate hypertrophy, the presence of subepithelial fibrosis has been described, as well as an association with symptom chronicity and little reversibility of the nasal airflow restriction.^43^ Remodeling was also demonstrated in the nasal mucosa of patients with mild and severe intensity persistent allergic rhinitis, although with relatively intact epithelium, characterized by intense collagen deposition in the superficial and submucosal layers, in addition to significant basement membrane thickening, which could reflect the high levels of TGF-β and its pro-fibrotic effect.^44^

Remodeling in allergic rhinitis, characterized by some degree of basement membrane thickening, without other alterations in the epithelium and submucosa,^45^ may be related to collagen deposition.^44^, ^46^ The allergic inflammation potentiates and accelerates this physiological process of collagen deposition in the basement membrane of the inferior turbinate.^7^, ^47^

The frequency of basement membrane thickening was similar between atopic (67%) and nonatopic (55%) subjects in our study. The proportion of eosinophils and neutrophils in the NL of atopic and non-atopic subjects was similar among those with BM thickening. On the other hand, the number of neutrophils in the nasal lavage of non-atopic subjects with basement membrane thickening was higher in comparison to NAT without BMT. This finding can associate the neutrophilic infiltration to the mucosal lesion mechanism in non-allergic patients. A similar number of eosinophils in the atopic and non-atopic NL samples, with basement membrane thickening, may also suggest the participation of eosinophils in the mucosal lesion of patients with allergic or non-allergic rhinitis.

Eosinophilic infiltration is the main cause of epithelial lesion, associated with loss of epithelial integrity in patients with allergic or non-allergic rhinitis.^6^, ^14^, ^29^, ^33^ In chronic neutrophilic and eosinophilic rhinosinusitis, remodeling findings were similar, which also corroborates the concept that the occurrence of mucosal remodeling is independent of the type of inflammatory reaction.^45^

## Conclusion

These findings show that in patients with rhinitis, allergic or non-allergic, there are some differences in the inflammatory process, but they may result in similar structural damage to the nasal mucosa. If there is an association between the progression of the inflammatory process in the nasal cavity lumen and the histopathological mucosal lesion (thickening of the basement membrane), this association is independent of the presence of atopy in patients with rhinitis.

## Conflicts of interest

The authors declare no conflicts of interest.
